# Individuals with severe visual field loss from stroke and glaucoma could have on‐road driving safety comparable to normally sighted drivers

**DOI:** 10.1111/aos.17512

**Published:** 2025-05-09

**Authors:** Tomas Bro, Jan Andersson

**Affiliations:** ^1^ Department of Biomedical and Clinical Sciences Linköping University Linköping Sweden; ^2^ Swedish National Road and Transport Research Institute Linköping Sweden

**Keywords:** driving, glaucoma, legislation, stroke, vision requirements, visual field loss

## Abstract

**Background:**

Vision is a critical component of safe driving, yet establishing effective legal vision requirements for driver licensing remains challenging. Current EU regulations mandate minimum standards for visual acuity and visual fields, but also allow exemptions based on practical driving tests. This study investigates the on‐road driving performance of individuals with visual field loss (VFL) who regained their licences after passing a simulator‐based assessment, compared to age‐matched and younger, normally sighted controls.

**Method:**

The study included 72 individuals with VFL who had successfully completed a simulator test and regained their driver's licences. Each participant was matched with an age‐ and gender‐matched normally sighted control and a younger normally sighted control (in total 212 participants). All participants underwent a standardized on‐road driving test administered by certified examiners blinded to group allocation. The test evaluated vehicle knowledge, eco‐driving, adherence to traffic rules and traffic safety/behaviour using the Swedish national driving test protocol. Logistic regression analysis was performed to assess factors influencing pass rates.

**Results:**

Participants with VFL achieved a pass rate of 68%, comparable to the age‐matched controls (66%) but lower than the younger controls (81%). No significant differences were observed in the proportions of passed tests, test elements, driving habits or interventions across groups. Within the VFL group, neither diagnosis type (e.g., glaucoma, stroke) nor the extent of visual field loss predicted test outcomes. While older groups (VFL and age‐matched controls) received more remarks regarding observational competence compared to younger controls, no differences emerged in risk identification or other competence areas.

**Conclusions:**

This study suggests that individuals with VFL can drive as safely as age‐matched, normally sighted controls. Simulator and on‐road tests are critical tools for individualized assessment, challenging the sole reliance on perimetry for licensing decisions. These findings support the inclusion of practical on‐road driving tests as a regulatory option for individuals with VFL, promoting mobility while maintaining road safety.

## 1 INTRODUCTION

Vision plays a crucial role in driving, and over the past decade, numerous studies have analysed the relationship between vision and road safety (Owsley et al., 2015). This research has been driven by a significant gap in understanding how legal vision requirements for driving licences should be designed. On one hand, vision requirements must be strict enough to exclude unsafe drivers. On the other hand, they should also enable safe drivers to retain their licences, as losing the ability to drive can have substantial consequences, including reduced mobility and a diminished quality of life (Nyberg et al., 2019). Despite extensive research, much remains unclear, making it challenging to establish objective criteria for adequate vision to ensure safe driving (Wood, 2019).

The current vision requirements for driving licences within the EU are governed by the Third European Driving Directive. This directive mandates a minimum binocular visual acuity of 0.5 decimal and a visual field extent of at least 120 degrees horizontally and 40 degrees vertically. However, the directive also allows for exemptions in exceptional cases, where drivers must undergo both a medical examination and a practical driving test (The Commission of the European Communities, 2009). While the directive provides a regulatory framework, member states have the autonomy to design their national legislation. As a result, both vision requirements and exemption policies vary across countries. In Sweden, legislation has historically been stricter than in other Nordic countries, with regulations based on threshold perimetry results (Bro & Lindblom, 2018).

In Sweden, medical doctors are obligated to notify the Swedish Transport Agency if a patient does not meet the medical requirements for driving (Körkortslagen, 1998, p. 488). For many conditions, such as dementia or psychiatric disorders, determining when this obligation applies can be ambiguous. For visual disorders, however, the cut‐off values are well defined.

In alignment with the European Driving Directive, Sweden developed an alternative assessment method using a simulator‐based practical driving test. This test was implemented by the Swedish National Road and Transport Research Institute (VTI). Between August 2016 and June 2018, VTI offered individuals who had lost their driver's licence due to visual field loss (VFL) the opportunity to take a simulator‐based driving test in Linköping at a cost of 1700 euros. Over 300 individuals participated, and approximately two‐thirds were subsequently able to apply to the Swedish Transport Agency to have their licences reinstated. Younger participants performed better than older ones, and individuals with peripheral loss outperformed those with central loss. Nevertheless, extensive VFL were often found to be compatible with safe driving (Bro & Andersson, 2022a, 2022b).

In June 2018, this testing was paused and has not resumed, partly due to concerns about the simulator's realism, such as its inability to replicate sharp curves. However, individuals who regained their licences through this process retained their exemptions. For those with progressive conditions such as glaucoma, updated visual field examinations must be submitted to the Swedish Transport Agency every two years to rule out deterioration.

This study serves as a follow‐up, using a practical on‐road driving test to assess individuals who regained their licences after passing the simulator test. Their performance was compared with that of two control groups of normally sighted individuals.

The primary aim of this study was to evaluate how well individuals with visual field loss, who regained their driver's licences based on an approved simulator test, would perform on a standard on‐road driving test. A secondary objective was to examine how the different VFL groups performed.

## 2 METHOD

### 2.1 Participants

The study was conducted by the Swedish National Road and Transport Research Institute (VTI) in Linköping, Sweden. In February 2022, data retrieved from the driving licence register identified 157 individuals with a valid Category B driving licence, which they had regained after passing a simulator test between 2018 and 2020. These individuals were contacted by mail and invited to undergo a practical driving test. They were informed that the test results would not affect their current driving licence status. Initially, 85 individuals expressed interest in participating. However, some were unable to take part due to deteriorating health or scheduling conflicts.

**FIGURE 1:**
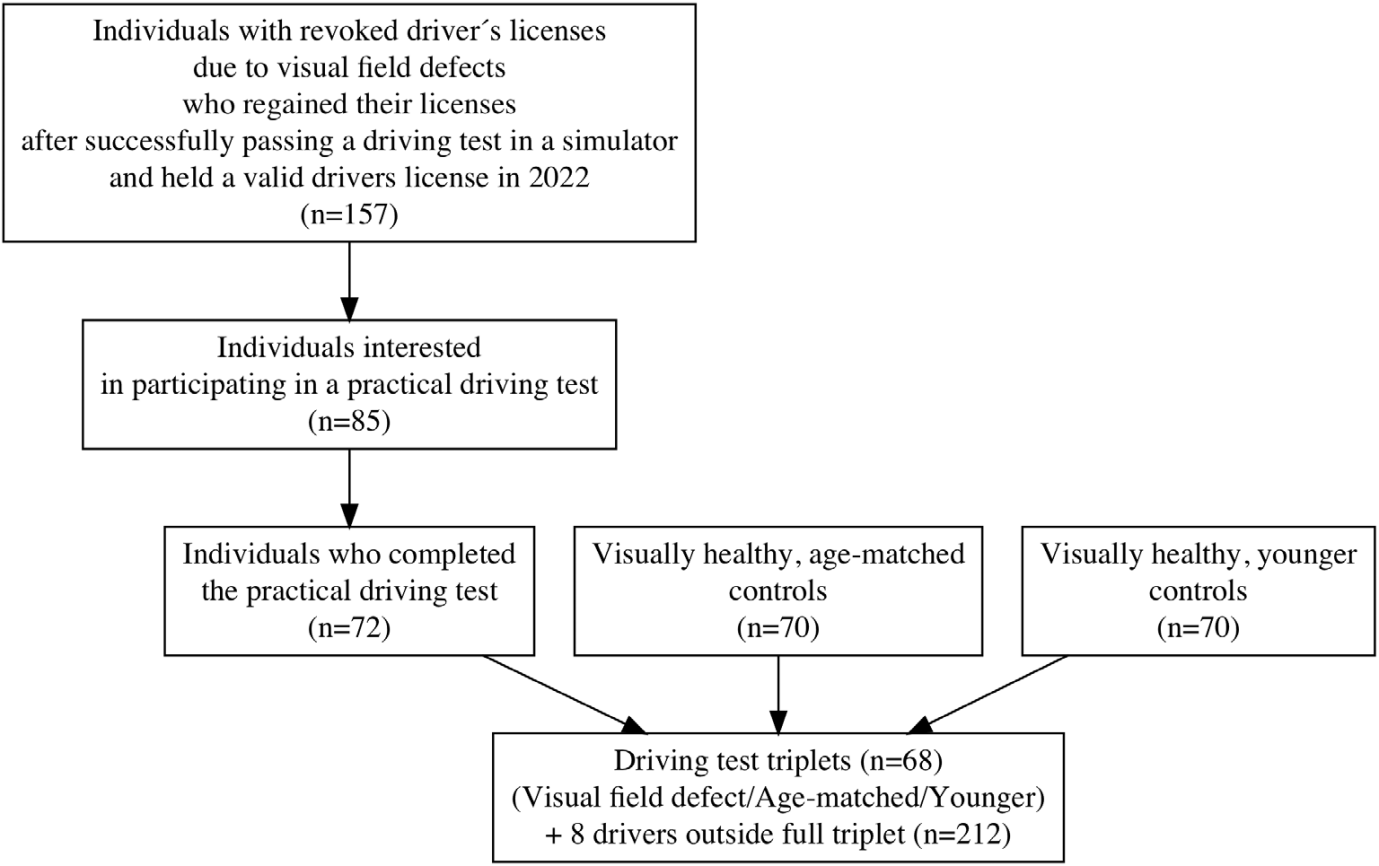
Flowchart of included participants.

For each participating individual with an exemption, a normally sighted control person of the same age, gender, driving experience and familiarity with the environment was recruited. Additionally, a younger control participant matched in gender and familiarity with the environment was included. Control participants were recruited through the Road Traffic Register, Facebook advertisements (in exceptional cases) and personal contacts. In total, 68 full triplets (each consisting of one participant with visual field loss, one age‐matched control and one younger control) were completed. An additional eight drivers participated outside of the full triplets, resulting in a total of 212 drivers (Figure 1). The time from regaining the licence to the practical driving test can be approximately described as between 2 years and 4 years. Ethical approval for the study was granted by the Linköping University Ethics Committee (Dnr 2014/124‐31).

### 2.2 Field of vision

None of the participants in the visual field loss group met the standards of an approved visual field defined by Swedish legislation (TSFS 2010:125, n.d.) (valid until January 31th 2025). This legislation requires that all corresponding test points (the maximum sensitivity for an overlapping location from two monocular perimetric examinations) within a 10° radius from the central fixation point must be at least 20 dB, and only one corresponding test point between a 10° and 20° radius from the central fixation point may be below 10 dB. Testing must be performed using Humphrey perimetry with a target size III or equivalent static threshold perimetry. In the peripheral visual field, no more than two adjacent missed points within a 120° × 40° area are permitted when tested with binocular supra‐threshold Esterman perimetry.

Visual field data used in the study were collected from the application to the simulator test. Since the cause for the revoked driver's licence prior to the simulator test could be either peripheral or central defects, not all individuals had available examinations from both supra‐threshold perimetry and threshold perimetry. However, one of these examinations was available for 93% of the participants with visual field loss. The analysis focused on the number of corresponding test points below 10 dB within 20° (out of 32) for central visual field with threshold perimetry and the total number of blind test points (out of 120, Esterman score) and within the 120° × 40° area (out of 64, defined by EU) (Bro, 2022) for peripheral visual field with supra‐threshold perimetry.

### 2.3 Driving test

Participants in a triplet (one with VFL, one age‐matched control and one younger control) completed the driving test in a dual‐brake vehicle provided by the Swedish Transport Administration (TRV). Testing took place in the same area and with the same driving examiner for all members of a triplet. The test followed the Swedish national standard for on‐road driving assessments required for a driver's licence. Thus, it was designed to evaluate general driving competence rather than specifically assessing how the driver adapted to impaired vision. Interventions, such as the examiner using the brake, were recorded.

A total of 39 traffic offices, spanning from northern to southern Sweden, participated in administering the tests. The tests within each triplet were conducted under similar conditions regarding route, season and time of day. The test began with a safety check followed by at least 25 min of driving in traffic. Examiners provided instructions to drive to a specified destination without pre‐determined routes, allowing them to adapt test content and repeat certain tasks if outcomes were unclear.

Examiners were blinded to the participants' group allocation (VFL or control) and participants were instructed not to reveal their visual status. Whether examiners were able to identify VFL participants regardless of blinding is unknown.

### 2.4 Instrument

The Swedish national driving test protocol includes 30 possible traffic situations, such as crossings, roundabouts, left turns, slippery surfaces and manoeuvring tasks like reversing, parking, or starting from an incline. During the test, the examiners marked which situations had been tested and if the driver had shown insufficient performance in the tested situation (remarks). Based on these observations, examiners thereafter made a holistic assessment focusing on four areas of competence: Vehicle knowledge/manoeuvring, Eco‐driving, Traffic rules, Traffic safety/behaviour. Each area is further classified into 1–7 subcategories. The protocol provides detailed descriptions of how tests should be administered, including instructions to participants and criteria for evaluating specific tasks (Trafikverket, 2023). Previous evaluations of the Swedish driving test's validity have shown high levels of inter‐rater agreement, with examiners and supervising examiners making the same decision in 93% of cases (Alger & Sundström, 2013).

### 2.5 Design

The study's primary objective was to assess differences in pass rates between the included groups. A secondary analysis examined whether the reasons for failing the test differed between groups. Within the VFL group, the causes of visual field impairment were analysed to determine whether these influenced the likelihood of passing the driving test. Finally, regression analyses were performed to identify factors associated with a passing result.

### 2.6 Statistical considerations

Statistical analyses included independent t‐tests for continuous variables and Fisher's exact test for categorical variables. Multivariate regression analysis was conducted to identify potential risk factors influencing a passing result. A significance level of *p* < 0.05 was applied throughout.

## 3 RESULTS

Individuals with visual field loss achieved a passing rate of 68%, compared to 66% in the age‐matched control group and 81% in the younger control group. None of these differences were significant with Fisher's exact test. The date range of tests was similar across all groups, with all assessments conducted during the warmer part of the year, ensuring that driving conditions were not influenced by snow or ice. Automatic and manual transmissions were equally distributed and consistent within each triplet, with no significant differences between the groups. Driving habits, the number of tested checkpoints, and interventions were also comparable across the three groups, with no statistically significant differences. Additionally, no significant differences were observed in how the tests were conducted, the number of tested situations or the frequency of interventions (Table 1). Except for interventions, tested and failed elements and situations, these findings remained consistent when comparing passed and failed drivers within each group (Table S1).

**TABLE 1:** Result of practical on‐road driving test for drivers with visual field defect compared to age‐matched and younger controls.

	Visual field loss, *N* = 72	Age‐matched control, *N* = 70	Younger control, *N* = 70	*p*‐Value VFL versus age‐matched, VFL versus younger, age‐matched versus younger
Approved	49 (68%)	46 (66%)	57 (81%)	0.9, 0.083, 0.054
Female gender	11 (15%)	10 (14%)	10 (14%)	>0.9, >0.9, >0.9
Age (SD)	66 (11)	67 (11)	26 (2)	0.7, <0.001, <0.001
Driven kilometres per year (SD)	15 329 (9653)	16 007 (11 578)	17 286 (14 407)	>0.9, 0.6, 0.6
Date range	2022‐04‐09 to 2022‐09‐27	2022‐05‐07 to 2022‐09‐27	2022‐06‐18 to 2022‐10‐19	
Manuel gear	32 (44%)	34 (49%)	31 (44%)	0.7, >0.9, 0.7
Tested situations mean (range)	14 (6–20)	13 (7–18)	14 (7–21)	0.6, 0.7, 0.4
Drivers with interventions	4 (5.6%)	3 (4.3%)	1 (1.4%)	>0.9, 0.4, 0.6

When examining different diagnoses within the visual field defect group, significant differences in age were noted. Participants with glaucoma had the highest mean age (71 years), followed by those with stroke (67 years) and other causes (58 years). Participants with stroke had a significantly higher number of blind test points on Esterman perimetry within the 120° × 40° visual field compared to those with other diagnoses. However, no significant differences were found in the proportion of approved tests, gender, driving habits or any other factors (Table 2). Except for interventions, tested and failed elements, and specific situations, no significant differences were observed for any factor when comparing passed and failed drivers within each visual field loss group (Table S2).

**TABLE 2:** Results of a practical on‐road driving test for drivers with visual field loss due to various causes.

	Stroke, *N* = 35	Glaucoma, *N* = 23	Other, *N* = 14	*p*‐Value stroke versus glaucoma, stroke versus other, glaucoma versus other
Approved	25 (71%)	14 (61%)	10 (71%)	0.6, >0.9, 0.7
Female gender	5 (14%)	4 (17%)	2 (14%)	>0.9, >0.9, >0.9
Age mean (SD)	67 (9)	71 (8)	58 (12)	0.070, 0.008, <0.001
Driven kilometres per year (D)	16 186 (10681)	13 667 (9465)	15 679 (7202)	0.3, 0.7, 0.3
Date range	2022‐06‐01 to 2022‐09‐22	2022‐04‐09 to 2022‐09‐27	2022‐06‐17 to 2022‐09‐27	NA, NA, NA
Manuel gear	14 (40%)	10 (43%)	8 (57%)	>0.9, 0.3, 0.5
Tested situations mean (range)	13 (8–19)	13 (6–20)	15 (11–17)	0.8, 0.056, 0.069
Drivers with interventions	3 (8.6%)	1 (4.3%)	0 (0%)	>0.9, 0.5, >0.9
Available supra threshold perimetry	26 (74%)	13 (57%)	8 (57%)	0.3, 0.3, >0.9
Esterman score mean (SD)	87 (10)	92 (9)	83 (12)	0.062, 0.4, 0.069
Blind test points within 120° × 40° mean (SD)	7.3 (5.8)	3.3 (3.3)	5.0 (4.2)	0.030, 0.4, 0.3
Available threshold perimetry	27 (77%)	22 (96%)	10 (71%)	0.073, 0.7, 0.057
Corresponding test points below 10 dB within 20° mean (SD)	6.0 (3.5)	4.5 (3.5)	4.6 (3.6)	0.080, 0.2, 0.8
Any available perimetry	32 (91%)	22 (96%)	13 (93%)	>0.9, >0.9, >0.9

**FIGURE 2:**
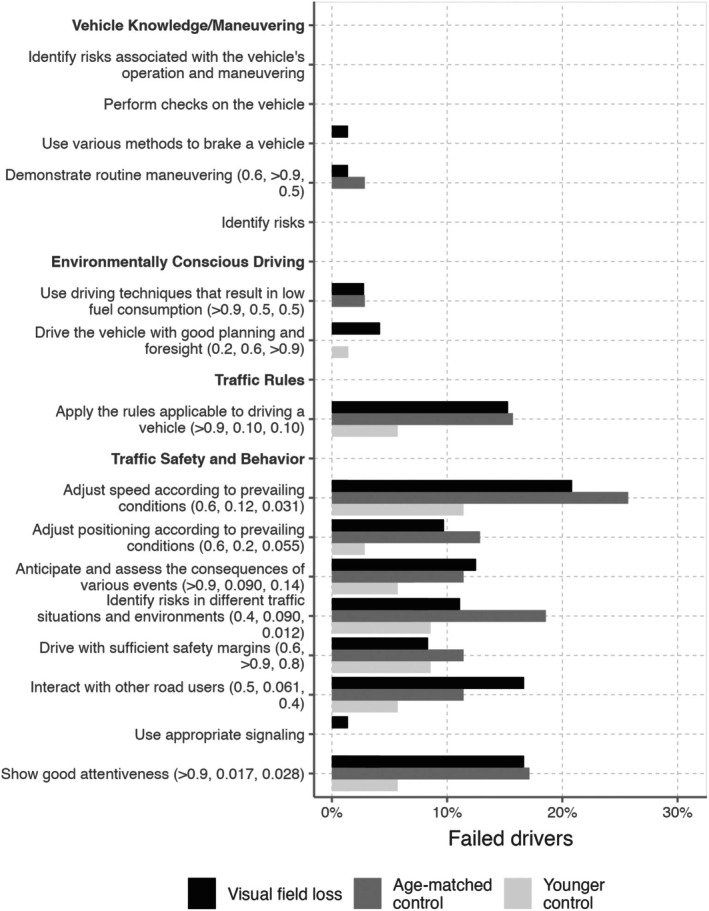
Proportion of drivers with remarks on different competence items (*p*‐values from Fisher's exact test are shown in parentheses for comparisons between drivers with visual field loss and age‐matched controls, drivers with visual field loss and younger controls, and age‐matched controls and younger controls.)

When analysing remarks in competence areas, no significant differences were found in good observation (showing good attentiveness) between the visual field defect group and the age‐matched control group. However, both of these groups had significantly more remarks in this area compared to the younger control group. Regarding the ability to identify risks in different traffic situations, the age‐matched control group (but not the visual field defect group) exhibited a significantly higher proportion of remarks compared to the younger group (*p* < 0.05, Fisher's exact test) (Figure 2).

In a multivariate logistic regression analysis, age was associated with a significantly lower odds ratio for passing the test (OR: 0.96–0.99; p < 0.05). In additional models focusing solely on individuals with visual field loss, no significant associations were found between specific diagnoses or the extent of peripheral or central visual field loss (Table 3).

**TABLE 3:** Multiple logistic regression analysis of factors suspected to influence the outcome of an approved practical on‐road driving test.

Group	Characteristic	OR	95% CI	*p*‐Value
All	Age	0.98	0.96, 0.99	0.008
	Female gender	0.47	0.20, 1.12	0.081
	Driven kilometres per year	1.00	1.00, 1.00	0.5
	Visual field loss	1.24	0.62, 2.53	0.5
Visual field loss diagnosis	Age	0.98	0.91, 1.03	0.4
	Female gender	1.51	0.31, 11.2	0.6
	Driven kilometres per year	1.00	1.00, 1.00	0.4
	Disease			
	Stroke			
	Glaucoma	0.82	0.25, 2.81	0.7
	Other	0.79	0.18, 3.78	0.8
Visual field loss extent	Age	1.01	0.94, 1.10	0.7
	Female gender	1.01	0.15, 8.69	>0.9
	Driven kilometres per year	1.00	1.00, 1.00	0.9
	Peripheral field (blind test points within 120° × 40°)	1.06	0.85, 1.32	0.6
	Central field (test points below 10 dB within 20°)	0.96	0.81, 1.15	0.7

## 4 DISCUSSION

This study on driving safety demonstrated that a selected group of individuals with severe visual field loss (VFL) was not rated as more unsafe than an age‐matched control group of drivers without visual field loss in an on‐road test. The test followed the standard protocol for ordinary driving licence assessments in Sweden. No significant differences were found between drivers with and without VFL in terms of failed competence areas outlined in the Swedish driving test protocol. Furthermore, regression models including age, gender and the presence or magnitude of visual field loss could not predict test failure.

To the best of our knowledge, this is the largest study to date comparing practical on‐road driving performance in individuals with VFL against normally sighted controls. A key strength of the study was the triplet design, where tests were conducted with matched participants under the same evaluator, who was blinded to the driver's visual status. However, the study also had notable limitations. Most importantly, visual field testing data were collected at the time of application for the simulator test, resulting in variability in the availability and recency of perimetric results. Some of these results were at least four years old. Nonetheless, individuals with progressive conditions were required to provide evidence of stable visual field results biennially after passing the simulator test.

Previous studies conducted in North America, Germany and Australia have compared on‐road driving safety between individuals with VFL and normally sighted controls. These studies employed evaluators ranging from driving instructors to occupational therapists and certified driver rehabilitation specialists, with testing protocols varying widely. The German studies, which used national driving test procedures similar to those in this study, involved fewer participants. Reported pass rates for drivers with VFL in these studies ranged from 20% to 88% (Table 4).

**TABLE 4:** Summary of previous studies on on‐road driving safety in individuals with visual field loss compared to normally sighted controls.

Author (year)	Country	*n* Case/controls (+*n* if several groups)	Diagnose	Evaluator (*n*)	Protocol	Approved case/controls
Kay et al. (2008)	Australia	20/80	Both visual field and acuity deficits	DI OT, OR (3)	“Standard assessment”	20%/84%
Silveira et al. (2007)	Australia	17/77	Esterman 120 degree criterion	DI in FS, OR in BS (2)	Driving performance	53%/96%
Wood et al. (2009)	USA	22 + 8/30	HA and QA	2 DRS in BS (2)	Manoeuvres and skills	73% + 88%/100%
Elgin et al. (2010)	USA	22 + 8/30	HA and QA	OT in FS (1)	10 manoeuvres, 6 skills, 5 grade scale	59–82%/90%
Parker et al. (2011)	USA	24/24	HA and QA	OT, DRS (2)	10 manoeuvres, 6 skills, 5 grade scale	88%/100%
Kasneci et al. (2014)	Germany	10 + 10/20	HA and glaucoma	UN in BS (1)	German national driving test	60% + 40%/80%
Bhorade et al. (2016)	USA	21/38	Glaucoma	DI in FS, DRS in BS (2)	Modified Washington University Road Test	48%/79%
Ungewiss et al. (2018)	Germany	10 + 10/10 + 10	Mixed	UN in BS (1)	German national driving test	60% + 40% /70% +100%
Bro et al. (2025) Present study	Sweden	72/70 + 70	Mixed	DI in FS	Swedish national driving test	68%/66% + 81%

Abbreviations: BS, back seat; DI, driving instructor; DRS, driver rehabilitation specialist; FS, front seat; G, glaucoma; HA, hemianopia; OR, orthoptist; OT, occupational therapist; QA, quadrantanopia; UN, unspecified evaluator.

The relationship between visual field scores on pass/fail test outcome in on‐road driving has previously been reported in data from Holland. Over one hundred drivers with a failed on‐road driving test were compared with as many drivers with a passed outcome, both groups having severe visual field loss. The Esterman visual field presented significantly increased odds for failing the driving test when more points were missed on a group level. However, at the same time, the Esterman visual field results could not predict the driving performance of the individual (Faraji et al., 2022). Our study could not confirm this finding but also had fewer participants in this analysis and did not have available Esterman perimetry from all drivers. It should also be noted that the Dutch dispensation protocol in this study did not assess general driving capacity but whether the candidate adapts to his or her field defect.

No previous research has demonstrated that individuals with VFL perform as well as or better than normally sighted, age‐matched controls. The most likely explanation for this finding in our study is the stringent selection process. The VFL group consisted of individuals who first opted to take an expensive simulator test, passed it and subsequently volunteered for the on‐road follow‐up test. Despite assurances that their results would not impact their driving licences, this group may have been highly motivated to demonstrate their competence. In contrast, the control groups, whose licences were never at risk, might not have been equally driven, though we lack data to confirm differences in motivation levels. Importantly, no significant differences in age, gender or diagnoses were found between those who regained their licences after passing the simulator test and those who participated in the on‐road study (Table S3).

The results clearly indicate that individuals with VFL can be safe drivers, performing at least as well as matched, normally sighted controls. These participants initially lost their licences based on perimetry results but regained them after passing a simulator test. Approximately 70% of individuals who took the Swedish simulator test passed, closely mirroring the pass rate in this on‐road study. The pass rate of 66% for normally sighted age‐matched controls raises the question of whether 34% really drive unsafely, or if the pass level is unnecessarily high. Our conclusion is that the Swedish driver test is a difficult test, as only approximately 50% normally sighted pass their first test when applying for a driver's licence in Sweden 2018–2021 (Sveriges Trafikutbildares Riksförbund, 2024).

In the simulator study, two independent raters assessed each participant, achieving 93% agreement (Bro & Andersson, 2022a). In a similar analysis of normally sighted participants, agreement between independent raters was 75% (Andersson, Andersson, et al., 2022). These findings highlight the challenge of rating driving ability, especially in borderline cases. This study demonstrates that while perimetry alone is insufficient to determine fitness to drive, on‐road tests are not infallible measures of driving ability either. Moreover, neither cognitive tests (Andersson & Peters, 2020) self‐assessments (Andersson, Bro, et al., 2022), nor perimetry (Bro & Andersson, 2022b) have reliably distinguished between safe and unsafe drivers. At present, a practical on‐road driving test appears to be the best available method.

In summary, individuals with severe visual field loss can be safe drivers. These findings support the implementation of individualized assessments to evaluate practical driving fitness in licensing decisions. An on‐road test conducted by a certified driving examiner remains the clinical gold standard. Based on this and other research, during 2023–2025 the Swedish Transport Agency is revising regulations to enable individuals who do not meet medical requirements to prove their driving competence through practical on‐road testing (Regeringskansliet, 2023). This regulatory update follows a directive from the Minister of Infrastructure to create a legal framework supporting such assessments.

## FUNDING INFORMATION

This study was funded by Stiftelsen Promobilia (Grant number 22014).

## Supporting information


Data S1.
