# Supplementary material for: Individuals with severe visual field loss from stroke and glaucoma could have on‐road driving safety comparable to normally sighted drivers
Source: Acta Ophthalmol. 2025 May 9;103(7):842–9. doi: 10.1111/aos.17512 (PMC12531607; doi:10.1111/aos.17512)
Supplement: Supplementary file 1 — Data S1. [file AOS-103-842-s001.docx]

Supplementary table 1. Results of the practical on-road driving test for drivers with visual field defects compared to age-matched and younger controls, categorized by approved or failed outcomes.

| Group | Visual field loss, n = 72 |  |  | Age-matched control n=70 |  |  | Young control =70 |  |  |
| --- | --- | --- | --- | --- | --- | --- | --- | --- | --- |
| Result | Approved, N = 49 (68%) | Failed, N = 23 (32%) | p-value | Approved, N = 46 (66%) | Failed, N = 24 (34%) | p-value | Approved, N = 57 (81%) | Failed, N = 13 (19%) | p-value |
| Female gender | 8 (16%) | 3 (13%) | >0.9 | 7 (15%) | 3 (12%) | >0.9 | 4 (7.0%) | 6 (46%) | 0.002 |
| Age mean (SD) | 66 (11) | 68 (9) | 0.6 | 66 (12) | 70 (9) | 0.2 | 27 (2) | 25 (2) | 0.077 |
| Driven kilometers per year | 14 837 (9 479) | 16 476 (10 191) | 0.4 | 15 578 (10 195) | 16 848 (14 116) | >0.9 | 17 518 (15 648) | 16 269 (7 034) | 0.5 |
| Date range | 2022-04-29 to 2022-09-27 | 2022-04-09 to 2022-09-27 |  | 2022-05-07 to 2022-09-27 | 2022-08-10 to 2022-09-26 |  | 2022-06-18 to 2022-10-19 | 2022-08-13 to 2022-09-27 |  |
| Manuel gear | 22 (45%) | 10 (43%) | >0.9 | 23 (50%) | 11 (46%) | 0.8 | 25 (44%) | 6 (46%) | >0.9 |
| Tested situations mean (range) | 14 (8-20) | 13 (6-17) | 0.017 | 14 (7-18) | 12 (7-18) | 0.12 | 14 (8-21) | 13 (7-16) | 0.6 |
| Failed situations mean (SD) | 0 (0-0) | 3 (0-12) | <0.001 | 0 (0-0) | 3 (0-6) | <0.001 | 0 (0-0) | 2 (0-4) | <0.001 |
| Failed competence elements mean (SD) | 0 (0-0) | 5 (2-9) | <0.001 | 0 (0-0) | 4 (1-9) | <0.001 | 0 (0-0) | 3 (0-5) | <0.001 |
| Interventions | 0 (0%) | 4 (17%) | 0.009 | 0 (0%) | 3 (12%) | 0.037 | 0 (0%) | 1 (7.7%) | 0.2 |
| Available supra threshold perimetry | 32 (65%) | 15 (65%) | >0.9 |  |  |  |  |  |  |
| Esterman score mean (SD) | 89 (10) | 85 (11) | 0.4 |  |  |  |  |  |  |
| Blind test points within 120°x40° mean (SD) | 5.6 (5.2) | 6.3 (5.3) | 0.7 |  |  |  |  |  |  |
| Available threshold perimetry | 38 (78%) | 21 (91%) | 0.2 |  |  |  |  |  |  |
| Corresponding test points below 10 dB within 20° mean (SD) | 5.5 (3.8) | 4.6 (3.0) | 0.5 |  |  |  |  |  |  |
| Any available perimetry | 45 (92%) | 22 (96%) | >0.9 |  |  |  |  |  |  |

Supplementary table 2. Results of the practical on-road driving test for drivers with visual field defects, categorized by diagnosis and approved/failed outcomes.

| Group | Stroke, n =35 |  |  | Glaucoma, n=23 |  |  | Other, n=14 |  |  |
| --- | --- | --- | --- | --- | --- | --- | --- | --- | --- |
| Result | Approved, N = 25 (71%) | Failed, N = 10 (29%) | p-value | Approved, N = 14 (61%) | Failed, N = 9 (39%) | p-value | Approved, N = 10 (71% | Failed, N = 4 (29%) | p-value |
| Female gender | 4 (16%) | 1 (10%) | >0.9 | 3 (21%) | 1 (11%) | >0.9 | 1 (10%) | 1 (25%) | 0.5 |
| Age mean (SD) | 66 (10) | 68 (8) | >0.9 | 70 (8) | 72 (9) | 0.5 | 57 (14) | 60 (4) | >0.9 |
| Driven kilometers per year | 15 160 (9 647) | 18 750 (13 137) | 0.4 | 14 179 (10 885) | 12 643 (6 329) | >0.9 | 14 950 (7 679) | 17 500 (6 455) | 0.5 |
| Date range | 2022-06-01 to 2022-09-21 | 2022-07-05 to 2022-09-22 |  | 2022-04-29 to 2022-09-27 | 2022-04-09 to 2022-09-20 |  | 2022-06-17 to 2022-09-12 | 2022-08-25 to 2022-09-27 |  |
| Manuel gear | 11 (44%) | 3 (30%) | 0.7 | 6 (43%) | 4 (44%) | >0.9 | 5 (50%) | 3 (75%) | 0.6 |
| Tested situations mean (range) | 14 (8-19) | 13 (10-16) | 0.3 | 14 (9-20) | 12 (6-15) | 0.033 | 15 (12-17) | 14 (11-17) | 0.6 |
| Failed situations mean (SD) | 0 (0-0) | 4 (2-12) | <0.001 | 0 (0-0) | 2 (0-4) | <0.001 | 0 (0-0) | 3 (2-4) | <0.001 |
| Failed competence elements mean (SD) | 0 (0-0) | 5 (2-9) | <0.001 | 0 (0-0) | 4 (2-5) | <0.001 | 0 (0-0) | 4 (2-4) | <0.001 |
| Interventions | 0 (0%) | 3 (30%) | 0.018 | 0 (0%) | 1 (11%) | 0.4 | 0 (0%) | 0 (0%) |  |
| Available supra threshold perimetry | 19 (76%) | 7 (70%) | 0.7 | 7 (50%) | 6 (67%) | 0.7 | 6 (60%) | 2 (50%) | >0.9 |
| Esterman score mean (SD) | 88 (10) | 84 (10) | 0.4 | 93 (9) | 91 (9) | 0.6 | 85 (11) | 74 (15) | 0.4 |
| Blind test points within 120°x40° mean (SD) | 6.7 (5.8) | 9.1 (5.6) | 0.2 | 3.14 (2.97) | 3.50 (3.89) | >0.9 | 5.17 (4.45) | 4.50 (4.95) | >0.9 |
| Available threshold perimetry | 18 (72%) | 9 (90%) | 0.4 | 13 (93%) | 9 (100%) | >0.9 | 7 (70%) | 3 (75%) | >0.9 |
| Corresponding test points below 10 dB within 20° mean (SD) | 5.67 (3.82) | 6.78 (2.99) | 0.4 | 5.46 (3.99) | 3.00 (2.06) | 0.2 | 5.29 (4.15) | 3.00 (1.00) | 0.4 |
| Any available perimetry | 22 (88%) | 10 (100%) | 0.5 | 13 (93%) | 9 (100%) | >0.9 | 10 (100%) | 3 (75%) | 0.3 |

Supplementary table 3. Comparison of individuals with visual field loss who passed a simulator test (2018–2020) and the subgroup who participated in the on-road study (2022).

|  | Passed simulator test, N = 157 | Completed practical driving test, N = 72 | p-value |
| --- | --- | --- | --- |
| Age (SD) | 64 (12) | 66 (11) | 0.2 |
| Female gender | 22 (14%) | 11 (15%) | 0.8 |
| Stroke | 93 (59%) | 35 (49%) | 0.2 |
| Glaucoma | 33 (21%) | 23 (32%) | 0.10 |
| Other | 31 (20%) | 14 (19%) | >0.9 |
